# 2-(4-Bromo­phen­yl)-2-oxoethyl 4-meth­oxy­benzoate

**DOI:** 10.1107/S1600536811018988

**Published:** 2011-05-25

**Authors:** Hoong-Kun Fun, Wan-Sin Loh, B. Garudachari, Arun M. Isloor, M. N. Satyanarayan

**Affiliations:** a X-ray Crystallography Unit, School of Physics, Universiti Sains Malaysia, 11800 USM, Penang, Malaysia; b Organic Chemistry Division, Department of Chemistry, National Institute of Technology-Karnataka, Surathkal, Mangalore 575 025, India; c Department of Physics, National Institute of Technology-Karnataka, Surathkal, Mangalore 575 025, India

## Abstract

In the title compound, C_16_H_13_BrO_4_, the benzene rings are almost perpendicular to each other, making a dihedral angle of 84.07 (8)°. In the crystal, the mol­ecules are linked into chains along the *a* axis *via* inter­molecular C—H⋯O hydrogen bonds. A C—H⋯π inter­action is also observed.

## Related literature

For background to and applications of phenacyl benzoates, see: Gandhi *et al.* (1995 ▶); Huang *et al.* (1996 ▶); Litera *et al.* (2006 ▶); Rather & Reid (1919 ▶); Ruzicka *et al.* (2002 ▶); Sheehan & Umezaw (1973 ▶). For the synthesis, see: Judefind & Reid (1920 ▶). For bond-length data, see: Allen *et al.* (1987 ▶).
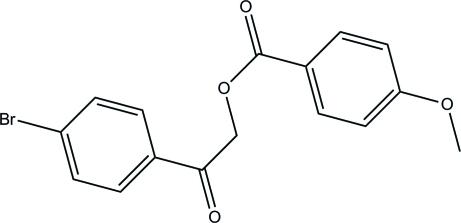


## Experimental

### 

#### Crystal data

C_16_H_13_BrO_4_
*M* *_r_* = 349.17
Triclinic, 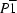

*a* = 7.9700 (5) Å
*b* = 7.9852 (5) Å
*c* = 11.3185 (7) Å
α = 86.536 (1)°
β = 83.205 (1)°
γ = 89.633 (1)°
*V* = 713.97 (8) Å^3^
*Z* = 2
Mo *K*α radiation
μ = 2.89 mm^−1^
*T* = 296 K
0.58 × 0.34 × 0.32 mm

#### Data collection

Bruker SMART APEXII DUO CCD area-detector diffractometer
Absorption correction: multi-scan (*SADABS*; Bruker, 2009 ▶) *T*_min_ = 0.286, *T*_max_ = 0.461
10671 measured reflections
3270 independent reflections
2767 reflections with *I* > 2σ(*I*)
*R*_int_ = 0.017

#### Refinement

*R*[*F* ^2^ > 2σ(*F* ^2^)] = 0.027
*wR*(*F* ^2^) = 0.069
*S* = 1.05
3270 reflections
191 parameters
H-atom parameters constrained
Δρ_max_ = 0.36 e Å^−3^
Δρ_min_ = −0.52 e Å^−3^

### 

Data collection: *APEX2* (Bruker, 2009 ▶); cell refinement: *SAINT* (Bruker, 2009 ▶); data reduction: *SAINT*; program(s) used to solve structure: *SHELXTL* (Sheldrick, 2008 ▶); program(s) used to refine structure: *SHELXTL*; molecular graphics: *SHELXTL*; software used to prepare material for publication: *SHELXTL* and *PLATON* (Spek, 2009 ▶).

## Supplementary Material

Crystal structure: contains datablocks global, I. DOI: 10.1107/S1600536811018988/is2716sup1.cif

Structure factors: contains datablocks I. DOI: 10.1107/S1600536811018988/is2716Isup2.hkl

Supplementary material file. DOI: 10.1107/S1600536811018988/is2716Isup3.cml

Additional supplementary materials:  crystallographic information; 3D view; checkCIF report

## Figures and Tables

**Table 1:** Hydrogen-bond geometry (Å, °) *Cg*1 is the centroid of the C1–C6 ring.

*D*—H⋯*A*	*D*—H	H⋯*A*	*D*⋯*A*	*D*—H⋯*A*
C2—H2*A*⋯O1^i^	0.93	2.48	3.386 (2)	164
C11—H11*A*⋯*Cg*1^ii^	0.93	2.81	3.6395 (18)	149

Symmetry codes: (i) 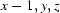; (ii) 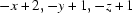.

**Fig. 1.**
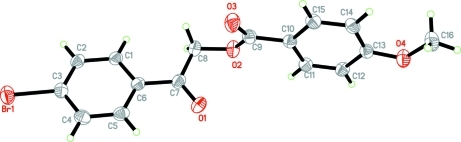
The molecular structure of the title compound, showing 30% probability displacement ellipsoids and the atom-numbering scheme.

**Fig. 2.**
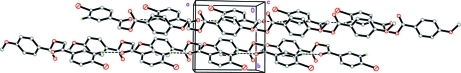
The crystal packing of the title compound, viewed along the c axis. H atoms not involved in the intermolecular interactions (dashed lines) have been omitted for clarity.

## supplementary crystallographic information

### Comment

Phenacyl benzoates are very useful protecting groups which can be easily removed by non-chemical reactions. The advantage of photosensitive blocking groups is that they can be removed under completely neutral and mild conditions (Sheehan & Umezaw, 1973; Ruzicka *et al.*, 2002; Litera *et al.*, 2006) used for identification of organic acids (Rather & Reid, 1919), synthesis of oxazoles, imidazoles (Huang *et al.*, 1996) and benzoxazepine (Gandhi *et al.*, 1995). Keeping this in view, we hereby report the crystal structure of 2-(4-bromophenyl)-2-oxoethyl 4-methoxybenzoate of potential commercial importance.

In the title compound (Fig. 1), the C1–C6 benzene ring [maximum deviation of 0.005 (2) Å at atom C6] is almost perpendicular with the C10–C15 benzene ring [maximum deviation of 0.014 (2) Å at atom C13] with a dihedral angle of 84.07 (8)°. Bond lengths (Allen *et al.*, 1987) and angles are within the normal ranges.

In the crystal packing (Fig. 2), the molecules are linked into chains along the *a* axis *via* intermolecular C2—H2A···O1 hydrogen bonds (Table 1). The crystal packing is further consolidated by C—H···π interactions, involving the centroids of C1–C6 phenyl ring.

### Experimental

The mixture of 4-methoxy benzoic acid (1.0 g, 0.0065 mol) sodium carbonate (0.766 g, 0.0072 mol) and 2-bromo-1-(4-bromophenyl)ethanone (2.00 g, 0.0072 mol) in dimethyl formamide (10 ml) was stirred at room temperature for 2 h. On cooling, the separated colourless needle-shaped crystals of 2-(4-bromophenyl)-2-oxoethyl 4-methoxybenzoate were collected by filtration. Compound was recrystallized from ethanol. Yield: 2.1 g (91.70%), *m.p.*: 429–430 K (Judefind & Reid, 1920).

### Refinement

All the H atoms were positioned geometrically and refined with a riding model with *U*_iso_(H) = 1.2 or 1.5*U*_eq_(C) (C—H = 0.93 or 0.96 Å). A rotating group model was applied to the methyl groups.

### Crystal data

**Table:**

C_16_H_13_BrO_4_	*Z* = 2
*M**_r_* = 349.17	*F*(000) = 352
Triclinic, *P*1	*D*_x_ = 1.624 Mg m^−^^3^
Hall symbol: -P 1	Mo *K*α radiation, λ = 0.71073 Å
*a* = 7.9700 (5) Å	Cell parameters from 4684 reflections
*b* = 7.9852 (5) Å	θ = 3.0–30.0°
*c* = 11.3185 (7) Å	µ = 2.89 mm^−^^1^
α = 86.536 (1)°	*T* = 296 K
β = 83.205 (1)°	Block, colourless
γ = 89.633 (1)°	0.58 × 0.34 × 0.32 mm
*V* = 713.97 (8) Å^3^	

### Data collection

**Table:**

Bruker SMART APEXII DUO CCD area-detector diffractometer	3270 independent reflections
Radiation source: fine-focus sealed tube	2767 reflections with *I* > 2σ(*I*)
graphite	*R*_int_ = 0.017
φ and ω scans	θ_max_ = 27.5°, θ_min_ = 1.8°
Absorption correction: multi-scan (*SADABS*; Bruker, 2009)	*h* = −10→10
*T*_min_ = 0.286, *T*_max_ = 0.461	*k* = −10→10
10671 measured reflections	*l* = −14→14

### Refinement

**Table:**

Refinement on *F*^2^	Primary atom site location: structure-invariant direct methods
Least-squares matrix: full	Secondary atom site location: difference Fourier map
*R*[*F*^2^ > 2σ(*F*^2^)] = 0.027	Hydrogen site location: inferred from neighbouring sites
*wR*(*F*^2^) = 0.069	H-atom parameters constrained
*S* = 1.05	*w* = 1/[σ^2^(*F*_o_^2^) + (0.0314*P*)^2^ + 0.2047*P*] where *P* = (*F*_o_^2^ + 2*F*_c_^2^)/3
3270 reflections	(Δ/σ)_max_ = 0.001
191 parameters	Δρ_max_ = 0.36 e Å^−^^3^
0 restraints	Δρ_min_ = −0.52 e Å^−^^3^

### Special details

**Table:**

Geometry. All e.s.d.'s (except the e.s.d. in the dihedral angle between two l.s. planes) are estimated using the full covariance matrix. The cell e.s.d.'s are taken into account individually in the estimation of e.s.d.'s in distances, angles and torsion angles; correlations between e.s.d.'s in cell parameters are only used when they are defined by crystal symmetry. An approximate (isotropic) treatment of cell e.s.d.'s is used for estimating e.s.d.'s involving l.s. planes.
Refinement. Refinement of *F*^2^ against ALL reflections. The weighted *R*-factor *wR* and goodness of fit *S* are based on *F*^2^, conventional *R*-factors *R* are based on *F*, with *F* set to zero for negative *F*^2^. The threshold expression of *F*^2^ > σ(*F*^2^) is used only for calculating *R*-factors(gt) *etc*. and is not relevant to the choice of reflections for refinement. *R*-factors based on *F*^2^ are statistically about twice as large as those based on *F*, and *R*- factors based on ALL data will be even larger.

### Fractional atomic coordinates and isotropic or equivalent isotropic displacement parameters (Å^2^)

**Table:**

	*x*	*y*	*z*	*U*_iso_*/*U*_eq_	
Br1	0.26530 (3)	0.93852 (3)	0.738521 (19)	0.06416 (9)	
O1	1.05611 (17)	0.7434 (2)	0.48016 (14)	0.0727 (4)	
O2	1.07533 (16)	0.58534 (16)	0.28028 (12)	0.0543 (3)	
O3	1.07066 (18)	0.84361 (18)	0.19107 (14)	0.0628 (4)	
O4	1.82176 (17)	0.60709 (18)	0.03308 (13)	0.0589 (3)	
C1	0.6023 (2)	0.7176 (2)	0.48878 (16)	0.0451 (4)	
H1A	0.5962	0.6522	0.4242	0.054*	
C2	0.4543 (2)	0.7690 (2)	0.55394 (16)	0.0471 (4)	
H2A	0.3495	0.7382	0.5337	0.057*	
C3	0.4655 (2)	0.8663 (2)	0.64896 (16)	0.0458 (4)	
C4	0.6205 (3)	0.9145 (2)	0.67953 (17)	0.0521 (4)	
H4A	0.6261	0.9816	0.7433	0.062*	
C5	0.7661 (2)	0.8622 (2)	0.61464 (17)	0.0502 (4)	
H5A	0.8705	0.8939	0.6351	0.060*	
C6	0.7594 (2)	0.7624 (2)	0.51864 (15)	0.0426 (4)	
C7	0.9203 (2)	0.7110 (2)	0.44998 (17)	0.0483 (4)	
C8	0.9105 (2)	0.6156 (3)	0.33964 (18)	0.0517 (4)	
H8A	0.8535	0.5094	0.3618	0.062*	
H8B	0.8449	0.6796	0.2858	0.062*	
C9	1.1450 (2)	0.7150 (2)	0.21027 (16)	0.0462 (4)	
C10	1.3205 (2)	0.6803 (2)	0.16089 (15)	0.0413 (4)	
C11	1.4168 (2)	0.5522 (2)	0.20810 (16)	0.0443 (4)	
H11A	1.3686	0.4808	0.2708	0.053*	
C12	1.5823 (2)	0.5308 (2)	0.16269 (17)	0.0464 (4)	
H12A	1.6453	0.4447	0.1946	0.056*	
C13	1.6566 (2)	0.6365 (2)	0.06941 (16)	0.0439 (4)	
C14	1.5604 (2)	0.7614 (2)	0.01947 (16)	0.0492 (4)	
H14A	1.6077	0.8305	−0.0448	0.059*	
C15	1.3943 (2)	0.7820 (2)	0.06586 (16)	0.0487 (4)	
H15A	1.3304	0.8663	0.0325	0.058*	
C16	1.9073 (3)	0.7168 (3)	−0.0585 (2)	0.0675 (6)	
H16A	2.0238	0.6843	−0.0727	0.101*	
H16B	1.9003	0.8299	−0.0341	0.101*	
H16C	1.8554	0.7100	−0.1304	0.101*	

### Atomic displacement parameters (Å^2^)

**Table:**

	*U*^11^	*U*^22^	*U*^33^	*U*^12^	*U*^13^	*U*^23^
Br1	0.06137 (14)	0.06633 (15)	0.05890 (14)	0.01421 (10)	0.01096 (9)	0.00724 (9)
O1	0.0370 (7)	0.1133 (13)	0.0702 (10)	−0.0055 (8)	−0.0113 (7)	−0.0132 (9)
O2	0.0440 (7)	0.0500 (7)	0.0654 (8)	0.0035 (5)	0.0050 (6)	0.0008 (6)
O3	0.0581 (8)	0.0542 (8)	0.0725 (9)	0.0160 (6)	0.0025 (7)	0.0021 (7)
O4	0.0462 (7)	0.0601 (8)	0.0664 (9)	0.0025 (6)	0.0045 (6)	0.0077 (7)
C1	0.0393 (8)	0.0515 (10)	0.0449 (9)	−0.0038 (7)	−0.0073 (7)	−0.0007 (7)
C2	0.0381 (8)	0.0516 (10)	0.0510 (10)	−0.0043 (7)	−0.0067 (7)	0.0056 (8)
C3	0.0470 (9)	0.0429 (9)	0.0442 (9)	0.0036 (7)	0.0014 (7)	0.0103 (7)
C4	0.0621 (11)	0.0497 (10)	0.0441 (10)	−0.0045 (8)	−0.0064 (8)	0.0000 (8)
C5	0.0470 (9)	0.0553 (11)	0.0489 (10)	−0.0104 (8)	−0.0114 (8)	0.0032 (8)
C6	0.0388 (8)	0.0468 (9)	0.0416 (9)	−0.0048 (7)	−0.0064 (7)	0.0069 (7)
C7	0.0370 (8)	0.0565 (11)	0.0505 (10)	−0.0035 (7)	−0.0067 (7)	0.0072 (8)
C8	0.0383 (9)	0.0569 (11)	0.0586 (11)	0.0000 (8)	−0.0005 (8)	−0.0037 (9)
C9	0.0481 (9)	0.0445 (9)	0.0462 (9)	0.0032 (7)	−0.0050 (7)	−0.0057 (7)
C10	0.0448 (9)	0.0388 (8)	0.0408 (9)	0.0015 (7)	−0.0050 (7)	−0.0058 (7)
C11	0.0463 (9)	0.0410 (9)	0.0448 (9)	−0.0028 (7)	−0.0049 (7)	0.0036 (7)
C12	0.0464 (9)	0.0416 (9)	0.0510 (10)	0.0008 (7)	−0.0091 (8)	0.0047 (7)
C13	0.0445 (9)	0.0430 (9)	0.0439 (9)	−0.0021 (7)	−0.0025 (7)	−0.0056 (7)
C14	0.0572 (11)	0.0451 (10)	0.0429 (9)	0.0013 (8)	0.0012 (8)	0.0030 (7)
C15	0.0563 (10)	0.0422 (9)	0.0465 (9)	0.0076 (8)	−0.0039 (8)	0.0018 (7)
C16	0.0556 (12)	0.0722 (14)	0.0686 (14)	−0.0042 (10)	0.0125 (10)	0.0063 (11)

### Geometric parameters (Å, °)

**Table:**

Br1—C3	1.8925 (18)	C7—C8	1.511 (3)
O1—C7	1.207 (2)	C8—H8A	0.9700
O2—C9	1.346 (2)	C8—H8B	0.9700
O2—C8	1.429 (2)	C9—C10	1.475 (2)
O3—C9	1.203 (2)	C10—C15	1.384 (3)
O4—C13	1.356 (2)	C10—C11	1.394 (2)
O4—C16	1.428 (2)	C11—C12	1.371 (2)
C1—C2	1.389 (2)	C11—H11A	0.9300
C1—C6	1.389 (2)	C12—C13	1.389 (2)
C1—H1A	0.9300	C12—H12A	0.9300
C2—C3	1.376 (3)	C13—C14	1.387 (3)
C2—H2A	0.9300	C14—C15	1.378 (3)
C3—C4	1.385 (3)	C14—H14A	0.9300
C4—C5	1.374 (3)	C15—H15A	0.9300
C4—H4A	0.9300	C16—H16A	0.9600
C5—C6	1.392 (3)	C16—H16B	0.9600
C5—H5A	0.9300	C16—H16C	0.9600
C6—C7	1.488 (2)		
			
C9—O2—C8	115.26 (14)	H8A—C8—H8B	108.0
C13—O4—C16	118.49 (15)	O3—C9—O2	123.12 (17)
C2—C1—C6	121.02 (17)	O3—C9—C10	124.67 (17)
C2—C1—H1A	119.5	O2—C9—C10	112.21 (14)
C6—C1—H1A	119.5	C15—C10—C11	118.55 (16)
C3—C2—C1	118.78 (16)	C15—C10—C9	118.67 (15)
C3—C2—H2A	120.6	C11—C10—C9	122.75 (16)
C1—C2—H2A	120.6	C12—C11—C10	120.37 (16)
C2—C3—C4	121.30 (17)	C12—C11—H11A	119.8
C2—C3—Br1	119.48 (14)	C10—C11—H11A	119.8
C4—C3—Br1	119.22 (14)	C11—C12—C13	120.61 (16)
C5—C4—C3	119.33 (18)	C11—C12—H12A	119.7
C5—C4—H4A	120.3	C13—C12—H12A	119.7
C3—C4—H4A	120.3	O4—C13—C14	124.61 (16)
C4—C5—C6	120.87 (17)	O4—C13—C12	115.91 (16)
C4—C5—H5A	119.6	C14—C13—C12	119.48 (16)
C6—C5—H5A	119.6	C15—C14—C13	119.44 (17)
C1—C6—C5	118.69 (17)	C15—C14—H14A	120.3
C1—C6—C7	122.38 (16)	C13—C14—H14A	120.3
C5—C6—C7	118.91 (16)	C14—C15—C10	121.49 (17)
O1—C7—C6	121.84 (18)	C14—C15—H15A	119.3
O1—C7—C8	119.99 (17)	C10—C15—H15A	119.3
C6—C7—C8	118.17 (15)	O4—C16—H16A	109.5
O2—C8—C7	111.04 (15)	O4—C16—H16B	109.5
O2—C8—H8A	109.4	H16A—C16—H16B	109.5
C7—C8—H8A	109.4	O4—C16—H16C	109.5
O2—C8—H8B	109.4	H16A—C16—H16C	109.5
C7—C8—H8B	109.4	H16B—C16—H16C	109.5
			
C6—C1—C2—C3	0.3 (3)	C8—O2—C9—C10	174.72 (15)
C1—C2—C3—C4	0.6 (3)	O3—C9—C10—C15	−15.5 (3)
C1—C2—C3—Br1	179.71 (13)	O2—C9—C10—C15	164.40 (16)
C2—C3—C4—C5	−0.9 (3)	O3—C9—C10—C11	162.56 (18)
Br1—C3—C4—C5	179.99 (14)	O2—C9—C10—C11	−17.5 (2)
C3—C4—C5—C6	0.3 (3)	C15—C10—C11—C12	1.5 (3)
C2—C1—C6—C5	−0.8 (3)	C9—C10—C11—C12	−176.60 (17)
C2—C1—C6—C7	−179.34 (17)	C10—C11—C12—C13	0.2 (3)
C4—C5—C6—C1	0.5 (3)	C16—O4—C13—C14	3.6 (3)
C4—C5—C6—C7	179.10 (17)	C16—O4—C13—C12	−176.97 (18)
C1—C6—C7—O1	−176.21 (19)	C11—C12—C13—O4	178.46 (17)
C5—C6—C7—O1	5.3 (3)	C11—C12—C13—C14	−2.1 (3)
C1—C6—C7—C8	3.8 (3)	O4—C13—C14—C15	−178.44 (17)
C5—C6—C7—C8	−174.72 (17)	C12—C13—C14—C15	2.2 (3)
C9—O2—C8—C7	−80.8 (2)	C13—C14—C15—C10	−0.4 (3)
O1—C7—C8—O2	−3.7 (3)	C11—C10—C15—C14	−1.4 (3)
C6—C7—C8—O2	176.31 (15)	C9—C10—C15—C14	176.76 (17)
C8—O2—C9—O3	−5.3 (3)		

### Hydrogen-bond geometry (Å, °)

**Table:**

*Cg*1 is the centroid of the C1–C6 ring.

**Table:**

*D*—H···*A*	*D*—H	H···*A*	*D*···*A*	*D*—H···*A*
C2—H2A···O1^i^	0.93	2.48	3.386 (2)	164
C11—H11A···Cg1^ii^	0.93	2.81	3.6395 (18)	149

Symmetry codes: (i) *x*−1, *y*, *z*; (ii) −*x*+2, −*y*+1, −*z*+1.
